# Prevascularization of collagen-glycosaminoglycan scaffolds: stromal vascular fraction versus adipose tissue-derived microvascular fragments

**DOI:** 10.1186/s13036-018-0118-3

**Published:** 2018-11-13

**Authors:** Thomas Später, Florian S. Frueh, Ruth M. Nickels, Michael D. Menger, Matthias W. Laschke

**Affiliations:** 1 Institute for Clinical & Experimental Surgery, Saarland University, 66421 Homburg/Saar, Germany; 2 Division of Plastic Surgery and Hand Surgery, University Hospital Zürich, University of Zürich, 8091 Zürich, Switzerland

**Keywords:** Tissue engineering, Stromal vascular fraction, Microvascular fragments, Integra®, Vascularization, Angiogenesis, Stem cells, Dorsal skinfold chamber

## Abstract

**Background:**

The seeding of scaffolds with the stromal vascular fraction (SVF) of adipose tissue is a common prevascularization strategy in tissue engineering. Alternatively, adipose tissue-derived microvascular fragments (ad-MVF) may serve as vascularization units. In contrast to SVF single cells, they represent a mixture of intact arteriolar, capillary and venular vessel segments. Therefore, we herein hypothesized that the ad-MVF-based prevascularization of scaffolds is superior to the conventional SVF single cells-based approach.

**Results:**

SVF single cells and ad-MVF were enzymatically isolated from epididymal fat pads of green fluorescent protein (GFP)^+^ donor mice to assess their viability and cellular composition using fluorescence microscopy and flow cytometry. Moreover, collagen-glycosaminoglycan matrices (Integra®) were seeded with identical amounts of the isolates and implanted into full-thickness skin defects within dorsal skinfold chambers of GFP^−^ recipient mice for the intravital fluorescent microscopic, histological and immunohistochemical analysis of implant vascularization and incorporation throughout an observation period of 2 weeks. Non-seeded matrices served as controls. While both isolates contained a comparable fraction of endothelial cells, perivascular cells, adipocytes and stem cells, ad-MVF exhibited a significantly higher viability. After in vivo implantation, the vascularization of ad-MVF-seeded scaffolds was improved when compared to SVF-seeded ones, as indicated by a significantly higher functional microvessel density. This was associated with an enhanced cellular infiltration, collagen content and density of CD31^+^/GFP^+^ microvessels particularly in the center of the implants, demonstrating a better incorporation into the surrounding host tissue. In contrast, non-seeded matrices exhibited a poor vascularization, incorporation and epithelialization over time.

**Conclusions:**

The present study demonstrates that ad-MVF are highly potent vascularization units that markedly accelerate and improve scaffold vascularization when compared to the SVF.

**Electronic supplementary material:**

The online version of this article (10.1186/s13036-018-0118-3) contains supplementary material, which is available to authorized users.

## Background

Tissue engineering is an interdisciplinary field of biomedical research focusing on the restoration of tissue defects or even on the replacement of complete organs [1–3]. A well-established approach for the generation of tissue constructs is the seeding of cells onto different biomaterials, which serve as three-dimensional scaffolds. To ideally promote the function and regenerative capacity of seeded cells, scaffolds should mimic the natural extracellular matrix [4–7]. Moreover, they should rapidly vascularize to ensure a sufficient oxygen supply and, thus, cellular survival [8–10]. To achieve this, prevascularization, i.e. the creation of preformed microvascular networks in scaffolds prior to their implantation, has emerged as a promising concept [11].

A common prevascularization strategy is the seeding of vessel-forming cells, such as endothelial cells or stem cells, onto scaffolds [12, 13]. However, blood vessels do not only consist of one specific cell type but exhibit a complex composition with an inner endothelial lining and surrounding vessel wall-stabilizing cell layers. Taking this into account, the stromal vascular fraction (SVF) of adipose tissue is frequently used to induce the formation of microvascular networks [10, 14]. The SVF results from the enzymatic digestion of fat samples and is a mixture of endothelial cells, pericytes, smooth muscle cells and stem cells [10, 14–16].

Due to its abundance and minimal-invasive accessibility, adipose tissue is not only an attractive source for the isolation of the SVF [17] but also for the harvesting of adipose tissue-derived microvascular fragments (ad-MVF) [18]. Digestion of adipose tissue for 45–60 min exclusively results in SVF single cells [19, 20], whereas a shorter digestion time of only 10 min provides a mixture of single cells and ad-MVF [21]. These ad-MVF still represent intact vessel segments and, thus, exhibit the unique feature of rapidly reassembling into new microvascular networks after transplantation [18]. Therefore, we herein hypothesized that the ad-MVF-based prevascularization of scaffolds is superior to the conventional SVF-based approach.

To test our hypothesis, we isolated both SVF single cells and ad-MVF from epididymal fat pads of donor mice according to well-established protocols. Subsequently, comparable amounts of the isolates were seeded onto collagen-glycosaminoglycan matrices. The seeded scaffolds were then implanted into full-thickness skin defects within mouse dorsal skinfold chambers for the in vivo analysis of implant vascularization and incorporation throughout an observation period of 2 weeks. Non-seeded matrices served as controls.

## Results

### Viability, cellular composition and activity of SVF single cells and ad-MVF

**Fig. 1:**
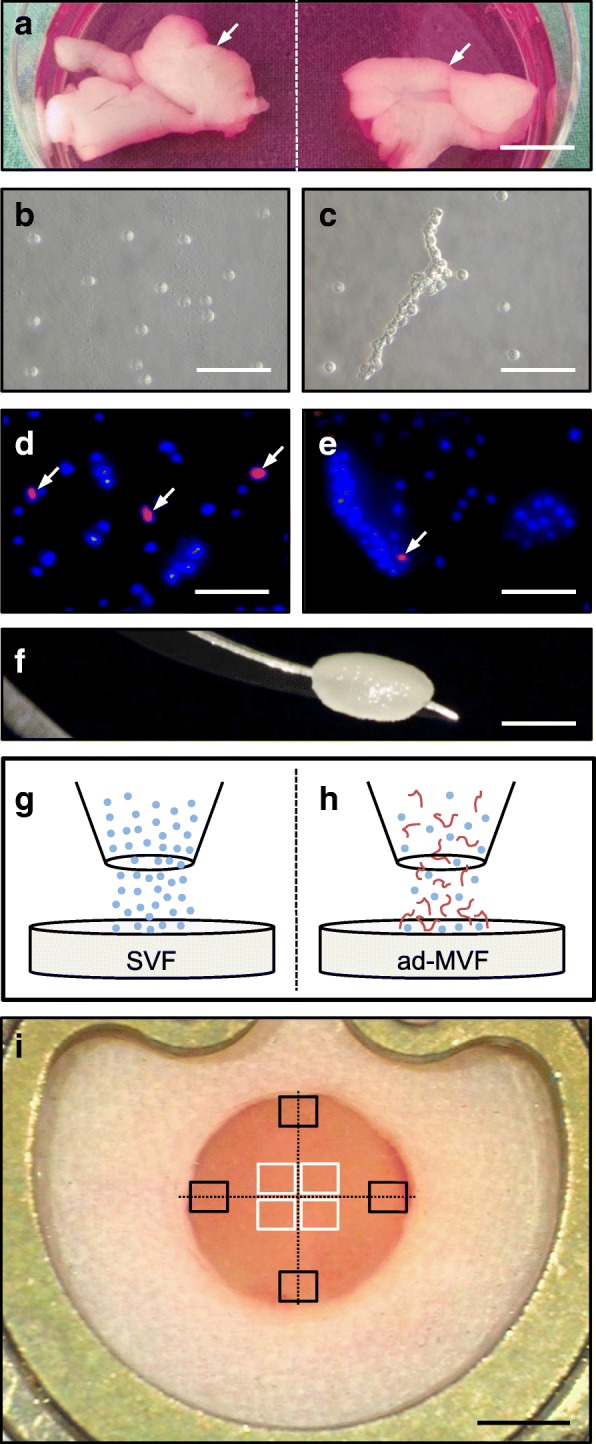
SVF and ad-MVF isolation and scaffold seeding. **a** Epididymal fat pads (arrows) of a GFP^+^ C57BL/6 donor mouse. Scale bar: 10 mm. **b**, **c** Freshly isolated SVF single cells (**b**) and ad-MVF (**c**). Scale bars: 50 μm. **d**, **e** Fluorescence microscopy of bisbenzimide (blue)/propidium iodide (red)-stained SVF single cells (**d**) and ad-MVF-associated cells (**e**) for the assessment of viability (arrows = dead propidium iodide^+^ cells). Scale bars: 50 μm. **f** Integra® scaffold on the tip of a micro forceps directly after sample preparation. Scale bar: 2.4 mm. **g**, **h** Scheme displaying scaffold seeding with SVF single cells (blue) (**g**) and ad-MVF (red) (**h**). **i** Overview of the dorsal skinfold chamber window directly after implantation of an Integra® scaffold (black frames = ROIs in the border; white frames = ROIs in the center). Scale bar: 1.8 mm

SVF single cells and ad-MVF were isolated from the bilateral epididymal fat pads of transgenic green fluorescent protein (GFP)^+^ C57BL/6 mice (Fig. 1a-c). This allowed the identification of both isolates after transplantation into GFP^−^ recipient animals. To analyze the viability of freshly isolated SVF single cells and ad-MVF, bisbenzimide/propidium iodide staining was performed to assess the percentage of propidium iodide^+^ dead cells in relation to all counted cells by means of fluorescence microscopy (Fig. 1d and e). This analysis revealed a viability of 82 ± 1% in the group of SVF single cells. In contrast, ad-MVF exhibited a significantly higher viability of 95 ± 1%. Additional flow cytometric analyses showed a comparable cellular composition of SVF single cells and ad-MVF (Table 1). They contained a mixture of CD31^+^ endothelial cells, α-smooth muscle actin (SMA)^+^ perivascular cells, adipocyte-specific adhesion molecule (ASAM)^+^ adipocytes as well as cells positive for the stromal/stem cell surface markers CD29, CD90 and CD117 (Table 1). To further assess the activity of isolated SVF single cells and ad-MVF in vitro, both isolates were cultivated over 6 days and analyzed by means of a water-soluble tetrazolium (WST)-1 assay directly after isolation as well as on day 3 and 6. The cellular activity of both isolates was comparable directly after isolation (absorbance 450 nm: 0.05 ± 0.02 (SVF) vs. 0.07 ± 0.03 (ad-MVF); *p* > 0.05) and progressively increased over time. Of interest, this increase was much more pronounced in the group of ad-MVF (absorbance 450 nm: 0.46 ± 0.15 (d3) and 1.18 ± 0.29 (d6)) when compared to SVF single cells (absorbance 450 nm: 0.06 ± 0.01 (d3) and 0.10 ± 0.02 (d6); *p* < 0.05).

**Table 1:** Cellular expression (%) of CD31, α-SMA, ASAM, CD29, CD90 and CD117 in the SVF and ad-MVF isolated from 1 mL epididymal fat pads of GFP^+^ donor mice (*n* = 3 per group), as assessed by flow cytometric analysis

	CD31	α-SMA	ASAM	CD29	CD90	CD117
SVF	26.4 ± 3.3	17.3 ± 1.2	14.4 ± 3.2	47.6 ± 4.7	10.4 ± 3.3	12.7 ± 3.3
Ad-MVF	18.0 ± 3.2	16.4 ± 2.3	13.0 ± 1.6	47.5 ± 4.7	7.1 ± 2.3	9.2 ± 1.7

Means ± SEM

### Seeding of scaffolds

As scaffold material we used Integra® dermal regeneration template single layer without silicone sheet (Fig. 1f), which is a ready-to-use porous off-the-shelf sheet matrix that consists of cross-linked bovine tendon collagen and shark glycosaminoglycan [22, 23]. To guarantee an equivalent overall number of cells per scaffold in both experimental groups, SVF single cells and ad-MVF were isolated from an identical volume of 250 μL adipose tissue. Quantitative analyses revealed that this volume corresponded to either ~ 1,000,000 SVF single cells or ~ 10,000 ad-MVF. Noteworthy, the ad-MVF isolates additionally contained ~ 200,000 single cells and, thus, have to be considered as a mixture of vessel segments and cells. For the seeding of the scaffolds (Figs 1g and h), the isolates were resuspended in 10 μL 0.9% NaCl. Importantly, the scaffold material absorbed the entire volume of both suspensions preventing the loss of individual cells and ad-MVF during the seeding procedure.

Histological analyses were performed to analyze the cell distribution within freshly seeded scaffolds. For this purpose, the number of cells was counted in 9 regions of interest (ROIs) per implant (equivalent to the entire implant area per section) to calculate the coefficient of variation (cv; standard deviation / mean) of the spatial cell distribution. Because ad-MVF were mainly trapped on the implants’ surface and SVF single cells were able to penetrate deeper into the pores of the scaffolds (Additional file 1: Figure S1a and b), the cv was significantly higher in the group of ad-MVF-seeded scaffolds when compared to SVF-seeded matrices (Additional file 1: Figure S1c).

### Vascularization of seeded scaffolds

**Fig. 2:**
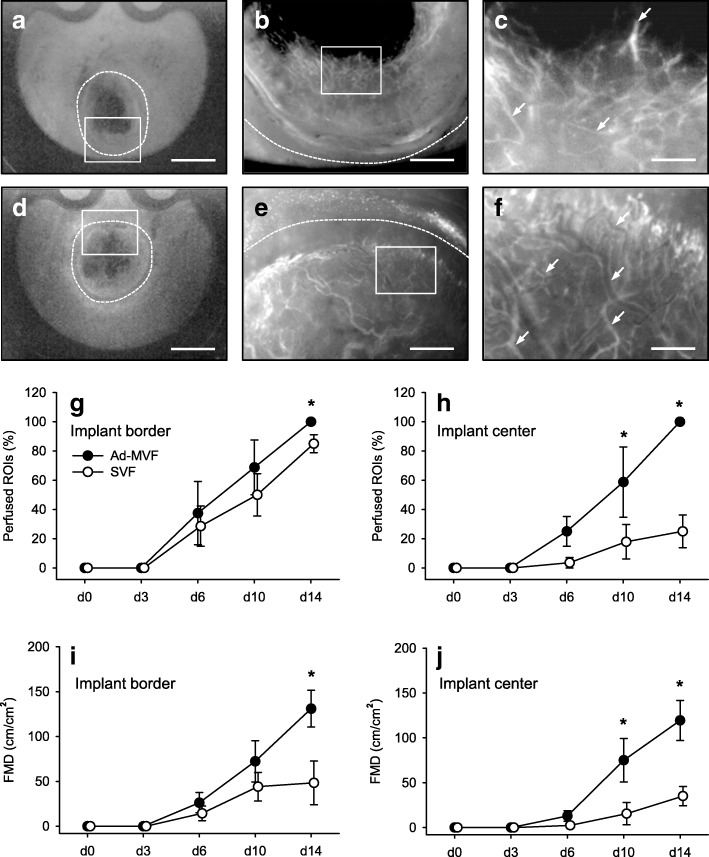
Intravital fluorescence microscopy of implanted scaffolds. **a-f** Intravital fluorescence microscopy (blue light epi-illumination with contrast enhancement by 5% FITC-labeled dextran) of SVF- (**a-c**) and ad-MVF-seeded (**d-f**) Integra® scaffolds on day 14 after implantation into full-thickness skin defects within dorsal skinfold chambers of C57BL/6 recipient mice (dotted lines = implant borders; arrows = perfused blood vessels; **b, e** = higher magnifications of inserts in **a** and **d**; **c, f** = higher magnifications of inserts in **b** and **e**). Scale bars: **a**, **d** = 2.4 mm; **b, e** = 500 μm; **c, f** = 125 μm. **g-j** Perfused ROIs (**g**, **h**) and FMD (**i**, **j**) in the border (**g**, **i**) and center zones (**h**, **j**) of SVF- (white circles, *n* = 8) and ad-MVF-seeded (black circles, *n* = 8) Integra® scaffolds on day 0, 3, 6, 10 and 14 after implantation, as assessed by intravital fluorescence microscopy. Means ± SEM. *p < 0.05 vs. SVF-seeded Integra® scaffolds

According to Sorg et al. [24, 25], a modified mouse dorsal skinfold chamber model was used to repetitively analyze the in vivo vascularization of SVF- and ad-MVF-seeded scaffolds by means of intravital fluorescence microscopy. The vascularization of the implanted scaffolds was assessed in 4 ROIs in their center and in 4 ROIs in their border zones (Fig. 1i). Functionality of individual microvessels was proven by their perfusion with the plasma marker fluorescein isothiocyanate (FITC)-labeled dextran (Fig. 2a-f). In both groups, the first blood-perfused microvessels could be detected in the border and center zones of the implants on day 6 (Fig. 2g-j). Throughout the following observation period, the density of these microvessels progressively increased (Fig. 2i and j). Notably, ad-MVF-seeded implants exhibited a significantly higher fraction of perfused ROIs and a higher functional microvessel density (FMD) in their border zones when compared to SVF-seeded scaffolds on day 14 after implantation (Fig. 2g and i). This difference in vascularization was even more pronounced in the center of the implants. Whereas ad-MVF-seeded scaffolds were completely vascularized on day 14 (corresponding to 100% perfused ROIs), the fraction of perfused ROIs was only 25% in the group of SVF-seeded implants (Fig. 2h). In addition, the FMD of ad-MVF-seeded scaffolds was also 3.4-fold higher at this time point (Fig. 2j).

The additional measurement of microhemodynamic parameters revealed that the diameter of individual microvessels within the border and center zones of the implants decreased in both groups over time (Table 2). This was associated with an increase of the centerline red blood cell (RBC) velocity and wall shear rate (Table 2). There were no significant differences of microhemodynamics within individual microvessels between SVF- and ad-MVF-seeded scaffolds throughout the 14-day observation period.

**Table 2:** Diameter, centerline RBC velocity and wall shear rate of individual microvessels within SVF- and ad-MVF-seeded Integra® matrices directly (0d) as well as 3, 6, 10 and 14 days after implantation into dorsal skinfold chambers

	0d	3d	6d	10d	14d
Diameter [μm]:					
SVF border	–	–	32.0 ± 2.6	18.3 ± 1.6	17.6 ± 1.4
ad-MVF border	–	–	27.4 ± 3.6	19.2 ± 0.8	17.6 ± 1.9
SVF center	–	–	30.6 ± 1.3	23.1 ± 2.9	15.7 ± 1.4
ad-MVF center	–	–	21.9 ± 9.9	22.8 ± 1.2	20.4 ± 3.2
Centerline RBC velocity [μm/s]:					
SVF border	–	–	299.8 ± 102.8	307.8 ± 71.7	657.7 ± 158.2
ad-MVF border	–	–	117.6 ± 47.3	284.7 ± 102.7	311.9 ± 51.8
SVF center	–	–	133.1 ± 84.1	388.2 ± 67.7	483.7 ± 82.0
ad-MVF center	–	–	182.3 ± 121.3	332.9 ± 113.5	309.8 ± 26.2
Wall shear rate [s^− 1^]:					
SVF border	–	–	75.2 ± 23.3	125.1 ± 20.3	291.8 ± 45.8
ad-MVF border	–	–	33.1 ± 11.9	116.8 ± 31.6	150.7 ± 31.9
SVF center	–	–	35.9 ± 23.6	145.9 ± 33.2	245.7 ± 55.5
ad-MVF center	–	–	58.5 ± 17.8	121.3 ± 44.6	130.5 ± 28.9

Means ± SEM

**Fig. 3:**
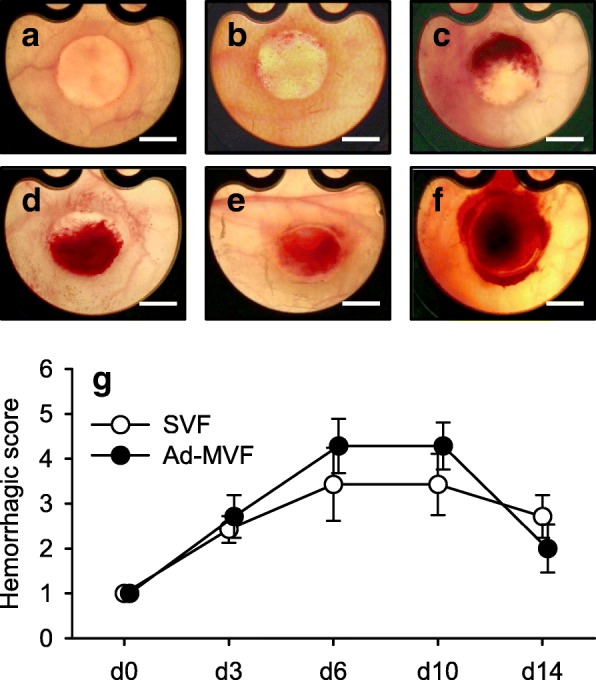
Implant-induced hemorrhage formation. **a-f** Transillumination stereomicroscopy of implanted ad-MVF-seeded Integra® scaffolds within dorsal skinfold chambers of C57BL/6 mice. The implants exhibit different degrees of implant-induced bleedings (% of total surface area) according to the semi-quantitative hemorrhagic score, i.e. 1: no bleeding (**a**), 2: 1–25% (**b**), 3: 26–50% (**c**), 4: 51–75% (**d**), 6: 76–100% (**e**), 6: bleeding exceeding implant surface (**f**). Scale bars: 2.3 mm. **g** Hemorrhagic score of SVF- (white circles, *n* = 8) and ad-MVF-seeded (black circles, *n* = 8) Integra® scaffolds on day 0, 3, 6, 10 and 14 after implantation, as assessed by stereomicroscopy. Means ± SEM

As previously reported [26, 27], the vascularization of ad-MVF-seeded implants induces hemorrhagic bleeding, which is most pronounced between day 6 and 10 of microvascular network formation. These findings were confirmed in the present study (Fig. 3a-g). The assessment of a hemorrhagic score showed no significant differences between SVF- and ad-MVF-seeded implants (Fig. 3g).

### Incorporation of SVF- and ad-MVF-seeded scaffolds

**Fig. 4:**
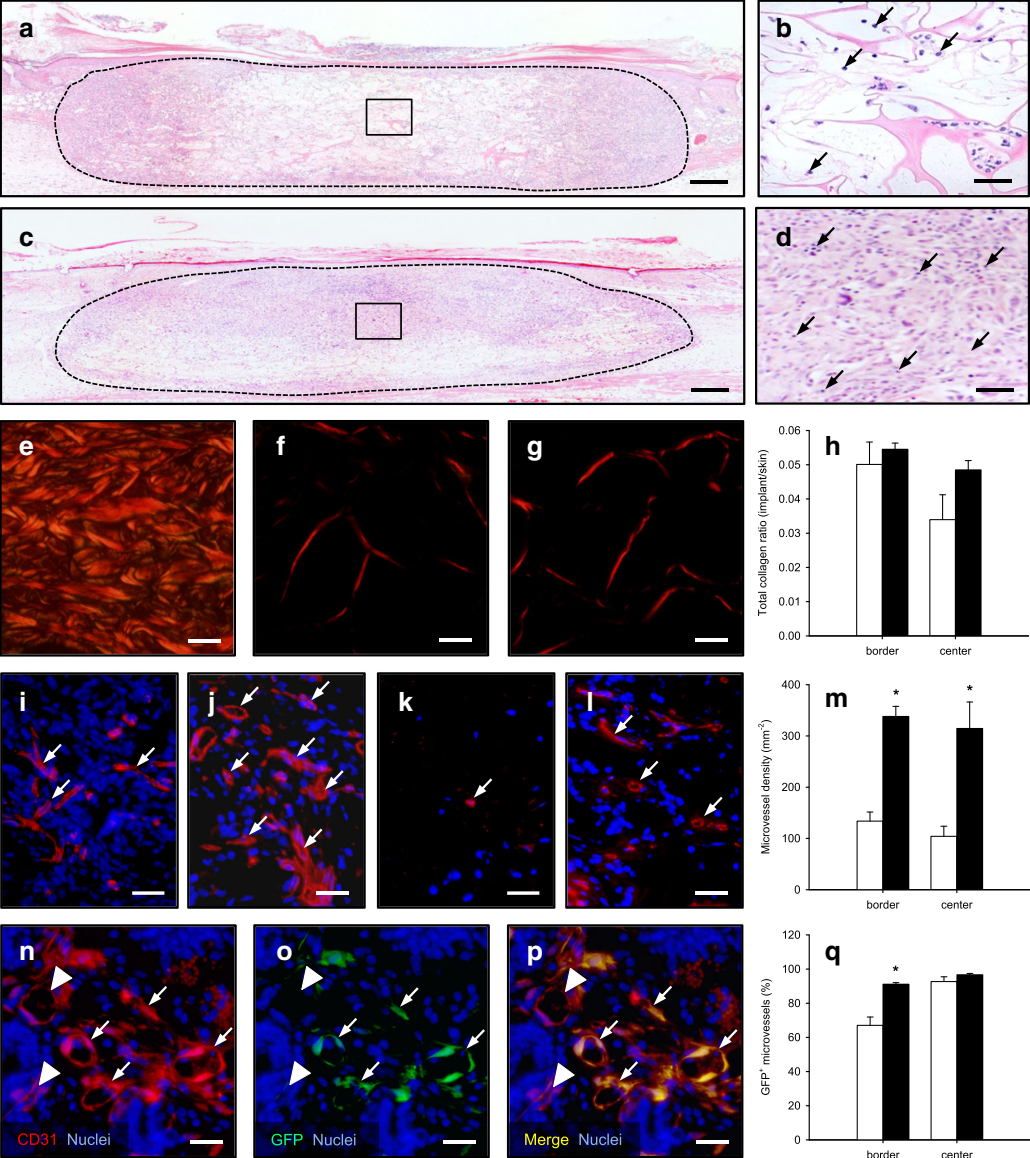
Incorporation and vascularization of implanted scaffolds. **a-d** HE-stained sections of SVF- (**a**, **b**) and ad-MVF-seeded (**c**, **d**) Integra® scaffolds on day 14 after implantation into full-thickness skin defects within dorsal skinfold chambers of C57BL/6 recipient mice (broken lines = implant; closed frames = center zones of the implants; **b**, **d** = higher magnifications of closed frames in **a** and **c** arrows = nuclei of individual cells). Scale bars: **a**, **c** = 260 μm; **b**, **d** = 40 μm. **e-g** Polarized light microscopy of Sirius red-stained sections of normal skin (**e**) as well as SVF- (**f**) and ad-MVF-seeded (**g**) Integra® scaffolds. Scale bars: 25 μm. **h** Total collagen ratio in the border and center zones of SVF- (white bars, *n* = 8) and ad-MVF-seeded (black bars, *n* = 8) Integra® scaffolds on day 14 after implantation, as assessed by histology. Means ± SEM. **i-l** Immunohistochemical detection of CD31^+^ microvessels (arrows) within the border (**i**, **j**) and center (**k**, **l**) zones of SVF- (**i**, **k**) and ad-MVF-seeded (**j, l**) Integra® scaffolds. Scale bars: 25 μm. **m** Microvessel density in the border and center zones of SVF- (white bars, *n* = 8) and ad-MVF-seeded (black bars, *n* = 8) Integra® scaffolds on day 14 after implantation, as assessed by immunohistochemistry. Means ± SEM. *p < 0.05 vs. SVF-seeded Integra® scaffolds. **n-p** Representative immunohistochemical staining of CD31^+^/GFP^+^ microvessels (arrows) and CD31^+^/GFP^−^ microvessels (arrowheads) within the border zone of a SVF-seeded Integra® scaffold on day 14 after implantation. Scale bars: 55 μm. **q** CD31^+^/GFP^+^ microvessels in the border and center zones of SVF- (white bars, *n* = 8) and ad-MVF-seeded (black bars, *n* = 8) Integra® scaffolds on day 14 after implantation, as assessed by immunohistochemistry. Means ± SEM. *p < 0.05 vs. SVF-seeded Integra® scaffolds

On day 14 after implantation, SVF- and ad-MVF-seeded scaffolds were additionally analyzed by means of histology and immunohistochemistry. The analysis of hematoxylin and eosin (HE)-stained sections showed a comparable infiltration of a dense granulation tissue at the border zones of SVF- (3697 ± 373 cells/mm^2^) and ad-MVF-seeded (4515 ± 347 cells/mm^2^) implants (Fig. 4a and c). In contrast, ad-MVF-seeded scaffolds exhibited a significantly higher number of infiltrating cells in their center zones (3465 ± 433 cells/mm^2^) when compared to SVF-seeded scaffolds (1884 ± 485 cells/mm^2^; *p* < 0.05) (Fig. 4b and d). Although not proven to be significant, further quantitative analyses of Sirius red-stained sections revealed that SVF-seeded implants contained less collagen fibers within their center zones when compared to ad-MVF-seeded scaffolds (Fig. 4e-h). Taken together, these results indicate an accelerated cellular infiltration and matrix formation in the center of ad-MVF-seeded scaffolds and, thus, an improved incorporation of the implants at the end of the 14-day observation period.

In line with our intravital fluorescent microscopic findings, additional immunohistochemical analyses showed a significantly higher density of CD31^+^ microvessels in both the border and center zones of ad-MVF-seeded scaffolds when compared to SVF-seeded implants (Fig. 4i-m). CD31^+^/GFP^+^ co-stainings further revealed that > 90% of all microvessels in the center zones of both implant types originated from the seeded GFP^+^ SVF or ad-MVF, respectively (Fig. 4n-q). However, whereas the border zones of ad-MVF-seeded scaffolds contained a comparably high fraction of GFP^+^ microvessels, the SVF-seeded implants exhibited a significantly reduced fraction of only 67% (Fig. 4q).

### Epithelialization of SVF- and ad-MVF-seeded scaffolds

**Fig. 5:**
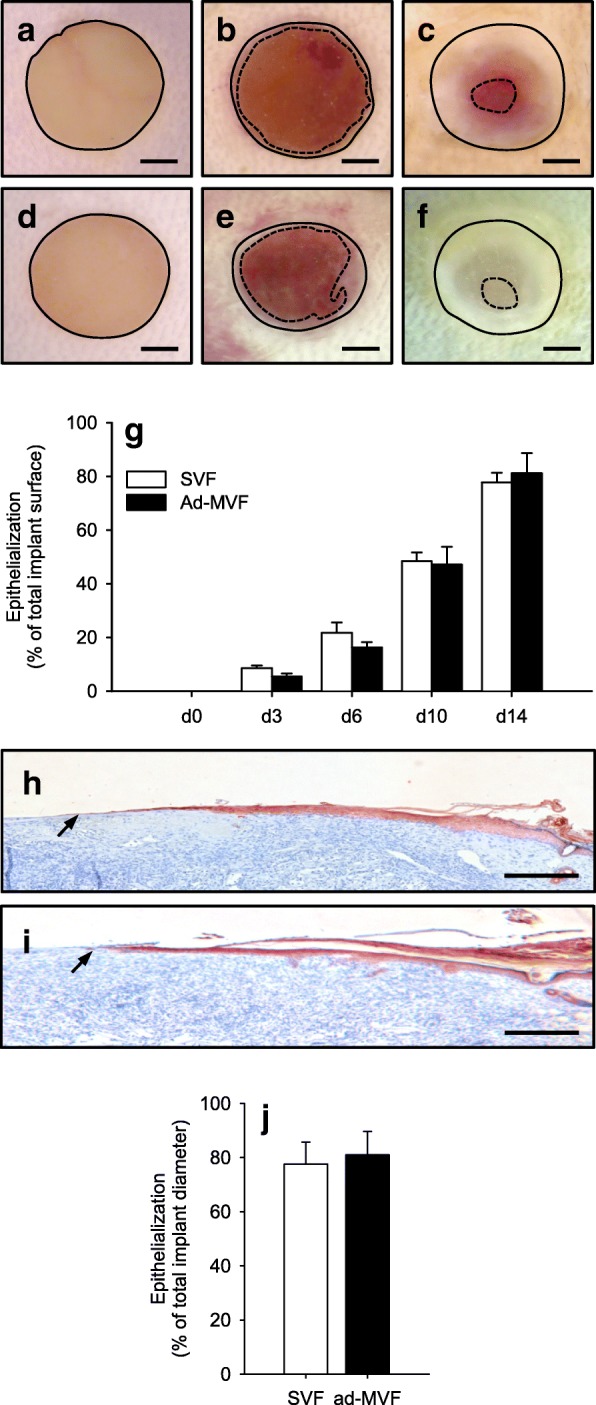
Epithelialization of implanted scaffolds. **a-f** Stereomicroscopic images showing the epithelialization of implanted SVF- (**a-c**) and ad-MVF-seeded (**d-f**) Integra® scaffolds directly (**a, d**) as well as on day 6 (**b**, **e**) and 14 (**c**, **f**) after implantation. Scale bars: 1 mm. **g** Epithelialization of SVF- (white bars, *n* = 8) and ad-MVF-seeded (black bars, *n* = 8) Integra® scaffolds on day 0, 3, 6, 10 and 14 after implantation, as assessed by trans-illumination microscopy. Means ± SEM. **h**, **i** Immunohistochemical detection of the cytokeratine^+^ epithelial layer (arrows) covering SVF- (**h**) and ad-MVF-seeded (**i**) Integra® scaffolds. Scale bars: 300 μm. **j** Epithelialization of SVF- (white bars, *n* = 8) and ad-MVF-seeded (black bars, *n* = 8) Integra® scaffolds on day 14 after implantation, as assessed by immunohistochemistry. Means ± SEM

The observation window of the dorsal skinfold chamber further allowed the repetitive stereomicroscopic analysis of implant epithelialization (Fig. 5a-f). Both SVF- and ad-MVF-seeded scaffolds exhibited a comparable epithelialization throughout the 14-day observation period (Fig. 5g). These results were confirmed by additional immunohistochemical analyses on day 14, which revealed that ~ 80% of SVF- and ad-MVF-seeded implants were covered with a cytokeratine^+^ epithelial layer (Fig. 5h-j).

### Vascularization, incorporation and epithelialization of non-seeded scaffolds

Although it was not the primary aim of the present study to compare seeded vs. non-seeded scaffolds, we additionally analyzed non-seeded Integra® matrices in the dorsal skinfold chamber model. By this, we could demonstrate that the vascularization, incorporation and epithelialization of SVF- and ad-MVF-seeded scaffolds were not markedly influenced by the implanted biomaterial itself. In fact, repetitive intravital fluorescence microscopy revealed a poor vascularization with < 15% perfused ROIs and a FMD of < 2 cm/cm^2^ in the implants’ border zones (Additional file 2: Figure S2a-c, d and f). The center of the scaffolds even remained avascular throughout the entire observation period (Additional file 2: Figure S2e and g). Additional histological analyses showed a poor cellular infiltration (border: 1817 ± 162 cell/mm^2^; center: 698 ± 43 cells/mm^2^) and collagen content of the implants on day 14 (Additional file 3: Figure S3a-c). The immunohistochemical detection of CD31^+^ microvessels further demonstrated a low microvessel density in the border and center zones of the non-seeded scaffolds (Additional file 3: Figure S3d). This was associated with a weak epithelialization (< 35%) of the implants’ surface on day 14 (Additional file 3: Figure S3e).

## Discussion

During the last two decades, a rapidly increasing number of studies have indicated a high vascularization potential of the SVF for applications in the field of tissue engineering and regenerative medicine [15, 28]. This heterogeneous cell isolate can easily be harvested from adipose tissue by enzymatic digestion without any complex cell separation or cultivation steps, which makes it an ideal candidate for autologous intra-operative one-step procedures. Accordingly, several automated and semi-automated SVF-isolating systems are already available for clinical use [29–32]. Of interest, others and we have shown that adipose tissue can also serve as a rich source for the isolation of intact and fully functional vessel segments when using a reduced enzymatic digestion time [21, 33]. These so-called ad-MVF rapidly reassemble into new microvascular networks after transplantation [34]. Importantly, our novel results now demonstrate that, due to this unique property, the seeding of scaffolds with ad-MVF markedly accelerates and improves vascularization after implantation when compared to the seeding with SVF.

In the present proof-of-principle study, we used the collagen-glycosaminoglycan matrix Integra®, which is a clinically well-established dermal substitute for the coverage of full-thickness skin defects. Beyond, Integra® is frequently used as scaffold material for experimental tissue engineering studies, because it easily enables cellular seeding due to its porous structure with pore sizes ranging between 20 and 125 μm [35]. In a previous study, we could show that a rapid and sufficient vascularization of 12.5 mm^2^ Integra® is achieved by a minimum seeding density of ~ 10,000 ad-MVF and 200,000 single cells, which can be isolated from 250 μL adipose tissue within a short enzymatic digestion time of 10 min [26]. The identical amount of adipose tissue was herein used for the generation of the SVF to ensure an identical cell load per scaffold, and, thus to guarantee a standardized comparison of the vascularization potential between the SVF and ad-MVF. For the complete disassembly of adipose tissue into the SVF a longer enzymatic digestion time was required. The longer exposure to collagenase may also explain the significantly reduced cell viability of the SVF when compared to ad-MVF. Noteworthy, our results are in line with previous studies reporting a SVF viability ranging between 80 and 83% [30, 36]. In addition, we detected a markedly higher cellular activity of ad-MVF on day 3 and 6 after isolation as a possible consequence of their improved viability. Moreover, the multi-cellular and three-dimensional architecture of ad-MVF may have further contributed to their higher cellular activity, as already described for cell spheroids [37]. This view is also supported by the observation that the vascularization of scaffolds is improved after their seeding with three-dimensional spheroids when compared to single cells [38].

All commercially available systems for the separation of the SVF from lipoaspirates exhibit a great heterogeneity in the outcome of the isolates’ cellular composition [30]. Along with this finding, our flow cytometric analyses revealed that the SVF and ad-MVF are a mixture of endothelial cells, perivascular cells, adipocytes and stem cells. Although this heterogeneous composition may complicate the standardization of the method for a broad clinical application, it also bears several major advantages. In fact, unlike purified cell preparations, the isolation of the SVF and ad-MVF is not associated with any complex cell separation or manipulative cultivation steps. This may facilitate the fulfilment of regulatory criteria for clinical approval. In addition, the mixture of different cell types may markedly contribute to an improved vascularization and tissue regeneration. In line with this view, animal studies reported a better therapeutic outcome in the treatment of acute myocardial infarction or nerve injury when transplanting the SVF in comparison to purified adipose-derived stem cells [39, 40].

For the in vivo analysis of vascularization and incorporation, we implanted non-seeded as well as SVF- and ad-MVF-seeded Integra® matrices into full-thickness skin defects within modified dorsal skinfold chambers. In combination with intravital fluorescence microscopy, this approach not only allowed us to detect newly formed microvessels within the implants, but also to prove their functionality and to assess microhemodynamic parameters by the direct visualization of blood perfusion. Of note, we found that non-seeded scaffolds only exhibited a poor vascularization and incorporation, indicating that the implanted biomaterial itself did not induce a strong host tissue response, as already shown in previous studies [41, 42].

In both SVF- and ad-MVF-seeded scaffolds, first blood-perfused microvessels could be found on day 6. At this time point, we also detected increased hemorrhage formation, which was most probably caused by an increased leakage of blood. As a typical sign of microvascular remodeling, microvessel diameters decreased while their centerline RBC velocities and wall shear rates increased throughout the further observation period. Although these characteristics of implant vascularization did not differ between the two groups, ad-MVF-seeded matrices exhibited a significantly higher number of perfused ROIs and a higher FMD in their border and center zones when compared to SVF-seeded implants. This can be explained by the fact that ad-MVF already represent fully functional arteriolar, capillary and venular segments that develop interconnections with each other and the surrounding microvasculature of the host tissue via the process of inosculation to establish a rapid blood perfusion within the implants [41]. In contrast, Koh et al. [43] showed that the endothelial cells within SVF suspensions first reassemble into new vessel channels as a precondition for the subsequent formation of microvascular networks. Hence, the time needed to complete this process is much longer when compared to ad-MVF-based vascularization. In this context, it should also be noted that ad-MVF exhibit a length up to 150 μm [26], preventing their homogeneous distribution within implants. On the other hand, a locally limited high density of rather large ad-MVF may facilitate the direct bridging of rather wide distances within tissue constructs in a short period. Accordingly, ad-MVF promote a more homogeneous blood perfusion within the border and center zones of implanted tissue constructs. In line with this view, the difference in vascularization between the SVF and ad-MVF group was most pronounced in the center of the implants, where > 90% of all detected microvessels originated from the seeded GFP^+^ SVF or ad-MVF.

Hypoxia-driven release of pro-angiogenic growth factors from the seeded cells may have additionally stimulated the angiogenic ingrowth of GFP^−^ microvessels from the surrounding host tissue. The physiological growth rate of microvessels is estimated not to be faster than ~ 5 μm/h [44]. Hence, such host vessels are mainly expected to occur in the border zones of the implants. In these regions, SVF- and ad-MVF-seeded scaffolds contained a fraction of ~ 30% and ~ 10% GFP^−^ microvessels, respectively. Taking into account that the microvessel density of ad-MVF-seeded scaffolds was ~ 3-fold higher, these immunohistochemical findings demonstrate a comparable number of ingrowing GFP^−^ microvessels from the surrounding host tissue into both implant types.

A sufficient vascularization represents a major prerequisite for an adequate implant incorporation [45, 46]. Accordingly, we herein observed an accelerated cellular infiltration and collagen formation in the center of ad-MVF-seeded Integra® matrices when compared to SVF-seeded ones. However, against our expectations, we did not detect any differences in implant epithelialization between the two groups. This may be due to the fact that both SVF- and ad-MVF-seeded matrices initially contained an identical cellular composition and load. Hence, they may have also released identical growth factor concentrations stimulating a vascularization-independent coverage with ingrowing keratinocytes from the wound edges.

## Conclusions

The present study demonstrates several important advantages of ad-MVF for the prevascularization of scaffolds when compared to the SVF. The faster isolation of ad-MVF may allow their application in a less time-consuming intra-operative procedure. Moreover, they exhibit a higher viability due the shorter enzymatic digestion time, which may markedly contribute to enhance cell yield and, thus, to reduce the volume of adipose tissue needed for individual patient treatments. Finally, ad-MVF are fully functional vessel segments that markedly accelerate and improve the vascularization of scaffolds. Taken together, all these unique features of ad-MVF suggest their future clinical use as vascularization units in tissue engineering.

## Methods

### Animals

Dorsal skinfold chambers were implanted in C57BL/6 wild-type mice (Institute for Clinical & Experimental Surgery, Saarland University, Homburg, Germany) with an age of 3–6 months and a body weight of 24–30 g. Epididymal fat was isolated from transgenic GFP^+^ mice (C57BL/6-Tg(CAG-EGFP)1Osb/J; The Jackson Laboratory, Bar Harbor, ME, USA) with an age of 7–12 months and a body weight of > 30 g. The animals were housed under a 12 h day/night cycle and fed with water and standard pellet food (Altromin, Lage, Germany) ad libitum.

### Isolation of SVF single cells and ad-MVF

For the isolation of ad-MVF, the fat pads were washed thrice in phosphate-buffered saline (PBS) before mechanical dissection. Subsequently, the minced adipose tissue was enzymatically digested with collagenase NB4G (0.5 U/mL; Serva, Heidelberg, Germany) under slow stirring and humidified atmospheric conditions (37 °C, 5% CO_2_) for 10 min. The digestion was neutralized with PBS supplemented with 20% fetal calf serum (FCS). Then, the cell-vessel suspension was incubated at 37 °C for 5 min and the fat supernatant was removed. The remaining suspension was filtered through a 500 μm mesh and a mixture of GFP^+^ ad-MVF and single cells was enriched to a final pellet by 5 min centrifugation at 120 x g.

For the isolation of SVF single cells, the bilateral fat pads from GFP^+^ C57BL/6 mice were harvested and mechanically minced as described above. The fat tissue was also enzymatically digested with collagenase NB4G (0.5 U/mL) under atmospheric conditions, however, for a longer time period of 60 min. To remove remaining fat clots, the suspension was then filtered through a 40 μm mesh and GFP^+^ single cells were subsequently enriched to a final pellet by a 5 min centrifugation at 120 x g.

### Viability of SVF single cells and ad-MVF

The final pellets of either SVF single cells or ad-MVF were resuspended in 1 mL PBS containing 2 mg/mL bisbenzimide and 1 mg/mL propidium iodide. Subsequently, 10 μL of these suspensions were transferred in a petri dish and analyzed by means of fluorescence microscopy. The analyses were performed in 10 randomly selected ROIs, each containing ~ 100 SVF single cells or ad-MVF cells.

### Flow cytometry

For flow cytometric analyses, isolated SVF single cells and ad-MVF, which were digested in Accutase® (BioLegend, Fell, Germany) for 30 min into single cells, were used. The single cells were analyzed for the expression of the monoclonal rat anti-mouse endothelial cell marker CD31-phycoerythrin (PE) (BD Biosciences, Heidelberg, Germany), the perivascular cell marker mouse anti-α-SMA (Thermo Fisher Scientific Inc., Waltham, MA, USA) and the monoclonal stromal/stem cell surface markers rat anti-mouse CD117-FITC (BD Biosciences), mouse anti-rat/mouse CD90-FITC (BioLegend) and hamster-anti-mouse CD29-FITC (BioLegend). Isotype identical rat IgG-PE or rat IgG-FITC (BD Biosciences), mouse IgG-FITC (BD Biosciences) and hamster IgG-FITC (BioLegend) served as controls. Additionally, cells were analyzed for the expression of the purified polyclonal sheep anti-mouse/human adipocyte marker ASAM (R&D Systems, Wiesbaden, Germany) followed by a secondary donkey anti-sheep IgG-Alexa488 antibody (Molecular Probes, Eugene, OR, USA). Flow cytometric analyses were performed by means of a FACScan (BD Biosciences) and data were assessed using the software package Cell-Quest Pro (BD Biosciences).

### Activity of SVF single cells and ad-MVF

For the in vitro analysis of cellular activity, final pellets of either SVF single cells or ad-MVF were cultivated in 96-well plates at 37 °C for 6 days in 100 μL Dulbecco’s Modified Eagle Medium (DMEM; 10% FCS, 100 U/mL penicillin, 0.1 mg/mL streptomycin; Biochrom, Berlin, Germany) under humidified conditions (5% CO_2_) with a medium change on day 3. The cellular activity of the isolates was assessed directly after isolation as well as on day 3 and 6 by means of a WST-1 assay (Roche diagnostics, Mannheim, Germany) according to the manufacturer’s instructions. For this purpose, 10 μL of WST-1 reagent were added per well and the plate was incubated for 30 min at 37 °C. Using a microplate reader, the absorption was measured at 450 nm with 620 nm set as a reference and the data were corrected to blank values.

### Seeding of scaffolds

For scaffold preparation, 12.6 mm^2^ samples were identically cut out of a 1.3 mm thick Integra® dermal regeneration template single layer without silicone sheet (Integra Life Sciences, Ratingen, Germany) with a 4 mm biopsy punch (kaiEurope GmbH, Solingen, Germany). The scaffolds were then placed on a 500 μm strainer for the seeding with ~ 1,000,000 SVF single cells or a mixture of ~ 10,000 ad-MVF and ~ 200,000 single cells, each resuspended in 10 μL 0.9% NaCl.

### Modified dorsal skinfold chamber model

Prior to the chamber implantation, the mice were anesthetized by an intraperitoneal injection of ketamine (75 mg/kg body weight; Ursotamin®, Serumwerke Bernburg, Bernburg, Germany) and xylazine (15 mg/kg body weight; Rompun®, Bayer, Leverkusen, Germany). As previously described in detail [47], the two symmetrical titanium frames of the chamber were then fixed on the extended dorsal skinfold of the animals. After the mice recovered for 48 h, a 4 mm full-thickness skin defect was created within the observation window of each chamber, using a dermal biopsy punch (kaiEurope GmbH) and microsurgical instruments. The defect was filled with a non-seeded, SVF- or ad-MVF-seeded scaffold and sealed with a removable cover glass.

### Stereomicroscopy

To measure both epithelialization and implant-induced bleeding of the implanted scaffolds by planimetry, the anesthetized animals were fixed on a Plexiglas® stage and the dorsal skinfold chambers were positioned under a stereomicroscope (Leica M651, Wetzlar, Germany) on day 0 (day of implantation), 3, 6, 10 and 14. Epithelialized and non-epithelialized implant areas (given in %) were visualized by epi-illumination and epithelialization was calculated by the equation: (total implant area – non-epithelialized implant area) / (total implant area) × 100 [27]. In addition, trans-illumination was used to determine the extent of implant-induced bleeding (given in % of implant surface) by means of a semi-quantitative hemorrhagic score as follows: 1: no bleeding, 2: 1–25%, 3: 26–50%, 4: 51–75%, 5: 76–100%, 6: bleeding exceeding implant surface. All microscopic images were recorded by a DVD system and analyzed using the computer-assisted off-line analysis system CapImage (Zeintl, Heidelberg, Germany).

### Intravital fluorescence microscopy

To achieve a sufficient contrast enhancement for intravital fluorescence microscopy, 0.1 mL of the blood plasma marker FITC-labeled dextran (5%; 150,000 Da; Sigma-Aldrich, Taufkirchen, Germany) was retrobulbarily injected into the venous plexus of the anesthetized animals. The observation window of the dorsal skinfold chamber was then positioned under a Zeiss Axiotech fluorescent microscope (Zeiss, Oberkochen, Germany) and the microscopic images were recorded by a charge-coupled device video camera (FK6990; Pieper, Schwerte, Germany) and a DVD system for the analysis by means of the computer-assisted off-line analysis system CapImage. As previously described [27], the vascularization of the implanted scaffolds was assessed in 4 ROIs in their center and in 4 ROIs in their border zones (Fig. 1i). ROIs exhibiting RBC-perfused microvessels were defined as perfused ROIs (% of all ROIs). In these ROIs the FMD was determined as the total length of all RBC-perfused microvessels per ROI (cm/cm^2^). Additionally, the diameter (d, μm) of 40 randomly selected microvessels was determined by the length of a measuring line perpendicular to the course of the vessels. Moreover, the centerline RBC velocity (v, μm/s) of the identical microvessels was assessed using the computer-assisted line-shift-diagram method [48]. These two parameters were subsequently used to calculate the wall shear rate (y, s^− 1^) by means of the Newtonian definition y = 8 x v/d.

### Experimental protocol

In a first set of in vitro experiments, epididymal fat pads were harvested from 12 GFP^+^ C57BL/6 donor mice to analyze the viability, composition and activity of SVF single cells and ad-MVF by means of fluorescence microscopy, flow cytometry and WST-1 assays. In a second set of in vitro experiments, SVF single cells and ad-MVF were harvested from 8 GFP^+^ C57BL/6 donor mice for the histological analysis of cell distribution within the matrices.

For in vivo analyses, SVF single cells and ad-MVF were isolated from the adipose tissue of 8 GFP^+^ C57BL/6 donor mice and subsequently seeded onto 16 Integra® scaffolds. The matrices were then implanted into full-thickness skin defects within dorsal skinfold chambers of 16 C57BL/6 wild-type mice. Vascularization, implant-induced bleeding and epithelialization of the implants (*n* = 8 per group) were analyzed by means of stereomicroscopy and intravital fluorescence microscopy on day 0 (day of implantation), 3, 6, 10 and 14. Vascularization and epithelialization of non-seeded Integra® scaffolds were further assessed in the dorsal skinfold chamber of 4 C57BL/6 wild-type mice. Thereafter, all animals were sacrificed by means of cervical dislocation and the dorsal skinfold chamber preparations were processed for histological and immunohistochemical analyses.

### Histology and immunohistochemistry

Formalin-fixed samples of non-seeded, SVF- and ad-MVF-seeded implants were embedded in paraffin and cut into 3 μm-thick sections. Individual sections were stained with HE according to standard procedures. By using a BX60 microscope (Olympus, Hamburg, Germany) and the imaging software cellCens Dimension 1.11 (Olympus), the density of infiltrating cells (mm^− 2^) was assessed in the border (2 ROIs) and center zones (3 ROIs) of the implants. In addition, Sirius red-stained sections were used to analyze the collagen content within the implants in relation to normal skin, as previously described in detail [41].

Sections were further co-stained with a monoclonal rat anti-mouse antibody against CD31 (1:100; Dianova, Hamburg, Germany) and a polyclonal goat antibody against GFP (1:200; Rockland Immunochemicals, Limerick, PA, USA), followed by a goat anti-rat IgG Alexa555 antibody (Life Technologies, Ober-Olm, Germany) and a biotinylated donkey anti-goat antibody (1:30; Dianova) as secondary antibodies. The biotinylated antibody was detected by streptavidin-Alexa 488 (1:50; Life Technologies) and cell nuclei were stained with Hoechst 33342 (2 μg/mL; Sigma-Aldrich). The density of CD31^+^ microvessels (given in mm^− 2^) and the fraction of CD31^+^/GFP^+^ microvessels (given in %) were quantitatively analyzed within the implants’ border and center zones.

For the immunohistochemical detection of the cytokeratine^+^ epithelial layer covering the implants on day 14, sections of the largest cross-sectional diameter of the scaffolds were incubated with a rabbit polyclonal anti-cytokeratine antibody (1:100; Abcam, Cambridge, UK) as primary antibody followed by a biotinylated goat anti-rabbit IgG antibody (ready-to-use; Abcam). The biotinylated antibody was detected by peroxidase-labeled-streptavidin (1:50; Sigma-Aldrich) and 3,3-diaminobenzidine (Sigma-Aldrich) was used as chromogen. Using a BZ-8000 microscopic system (Keyence, Osaka, Japan), the length of the cytokeratine^+^ epithelial layer and the diameter of the implants were measured to assess epithelialization as: (length of cytokeratine^+^ epithelial layer / total diameter of implant) * 100.

For the in vitro analysis of cell distribution, freshly SVF- and ad-MVF-seeded scaffolds were embedded in Tissue-tek® O.C.T. compound (A. Hartenstein GmbH, Würzburg, Germany), quick-frozen in liquid nitrogen at − 196 °C and subsequently cut into 3 μm-thick cryosections. Cell nuclei were stained with Hoechst 33342 (2 μg/mL; Sigma-Aldrich) and the sections were examined with a BX60 microscope (Olympus). The number of cells was determined in 9 ROIs per implant (equivalent to the entire implant area per section) to calculate the cv (standard deviation / mean) of the spatial cell distribution.

### Statistical analysis

After testing the data for normal distribution and equal variance, differences between the groups were analyzed by the unpaired Student’s t-test (SigmaPlot 11.0; Jandel Corporation, San Rafael, CA, USA). In case of non-parametric data, a Mann-Whitney rank sum test was used. All values are expressed as means ± SEM. Statistical significance was accepted for a value of *p* < 0.05.

## Additional files

Additional file 1: **Figure S1.** Cell distribution within SVF- and ad-MVF-seeded scaffolds. **a, b** Detection of cell nuclei (arrows) within a SVF- (**a**) and an ad-MVF-seeded (**b**) Integra® scaffold (dotted lines = implant border; green signals = autofluorescence of the biomaterial). Scale bars: 150 μm. **c** Cv of SVF- (white bar, *n* = 4) and ad-MVF-seeded (black bar, n = 4) Integra® scaffolds directly after the seeding procedure, as assessed by histology. Means ± SEM. *p < 0.05 vs. SVF-seeded Integra® scaffolds. (PPTX 849 kb)

Additional file 2: **Figure S2.** Intravital fluorescence microscopy of implanted non-seeded scaffolds. **a-c** Intravital fluorescence microscopy (blue light epi-illumination with contrast enhancement by 5% FITC-labeled dextran) of a non-seeded Integra® scaffold on day 14 after implantation into a full-thickness skin defect within the dorsal skinfold chamber of a C57BL/6 recipient mouse (dotted lines = implant borders; arrows = perfused blood vessels; **b, c** = higher magnifications of inserts in **a** and **b**). Scale bars: **a** = 2.4 mm; **b** = 500 μm; **c** = 125 μm. **d-g** Perfused ROIs (**d, e**) and FMD (**f, g**) in the border (**d, f**) and center zones (**e, g**) of non-seeded Integra® scaffolds (grey circles, *n* = 4) on day 0, 3, 6, 10 and 14 after implantation, as assessed by intravital fluorescence microscopy. Means ± SEM. (PPTX 651 kb)

Additional file 3: **Figure S3.** Incorporation, vascularization and epithelialization of implanted non-seeded scaffolds. **a, b** HE-stained section of a non-seeded Integra® scaffold on day 14 after implantation into a full-thickness skin defect within the dorsal skinfold chamber of a C57BL/6 recipient mouse (broken line = implant; closed frame = center zone of the implant; **b** = higher magnification of closed frame in **a**; arrows = nuclei of individual cells). Scale bars: **a** = 260 μm; **b** = 40 μm. **c** Total collagen ratio in the border and center zones of non-seeded Integra® scaffolds (grey bars, *n* = 4) on day 14 after implantation, as assessed by histology. Means ± SEM. **d** Microvessel density in the border and center zones of non-seeded Integra® scaffolds (grey bars, *n* = 4) on day 14 after implantation, as assessed by immunohistochemistry. Means ± SEM. **e** Epithelialization of non-seeded Integra® scaffolds (grey circles, *n* = 4) on day 0, 3, 6, 10 and 14 after implantation, as assessed by trans-illumination microscopy. Means ± SEM. (PPTX 1734 kb)

## Abbreviations

Ad-MVF: Adipose tissue-derived microvascular fragments
ASAM: Adipocyte-specific adhesion molecule
cv: Coefficient of variation
FCS: Fetal calf serum
FITC: Fluorescein-isothiocyanate
FMD: Functional microvessel density
GFP: Green fluorescent protein
HE: Hematoxylin and eosin
PBS: Phosphate-buffered saline
PE: Phycoerythrin
RBC: Red blood cell
ROI: Region of interest
SVF: Stromal vascular fraction
α-SMA: α-Smooth muscle actin

## References

[1] Griffith LG, Naughton G (2002). Tissue engineering-current challenges and expanding opportunities. Science.

[2] Metcalfe AD, Ferguson MW (2007). Tissue engineering of replacement skin: the crossroads of biomaterials, wound healing, embryonic development, stem cells and regeneration. J R Soc Interface.

[3] Tiranathanagul K, Dhawan V, Lytle IF, Zhang W, Borschel GH, Buffington DA (2007). Tissue engineering of an implantable bioartificial hemofilter. ASAIO J.

[4] Wisser D, Steffes J (2003). Skin replacement with a collagen based dermal substitute, autologous keratinocytes and fibroblasts in burn trauma. Burns.

[5] Hutmacher DW, Cool S (2007). Concepts of scaffold-based tissue engineering - the rationale to use solid free-form fabrication techniques. J Cell Mol Med.

[6] Hutmacher DW, Sittinger M, Risbud MV (2004). Scaffold-based tissue engineering: rationale for computer-aided design and solid free-form fabrication systems. Trends Biotechnol.

[7] Trottier V, Marceau-Fortier G, Germain L, Vincent C, Fradette J (2008). IFATS collection: using human adipose-derived stem/stromal cells for the production of new skin substitutes. Stem Cells.

[8] Levenberg S, Rouwkema J, Macdonald M, Garfein ES, Kohane DS, Darland DC (2005). Engineering vascularized skeletal muscle tissue. Nat Biotechnol.

[9] Blinder YJ, Freiman A, Raindel N, Mooney DJ, Levenberg S (2015). Vasculogenic dynamics in 3D engineered tissue constructs. Sci Rep.

[10] Cerino G, Gaudiello E, Muraro MG, Eckstein F, Martin I, Scherberich A (2017). Engineering of an angiogenic niche by perfusion culture of adipose-derived stromal vascular fraction cells. Sci Rep.

[11] Laschke MW, Menger MD (2016). Prevascularization in tissue engineering: current concepts and future directions. Biotechnol Adv.

[12] Stosich MS, Bastian B, Marion NW, Clark PA, Reilly G, Mao JJ (2007). Vascularized adipose tissue grafts from human mesenchymal stem cells with bioactive cues and microchannel conduits. Tissue Eng.

[13] Seebach C, Henrich D, Kähling C, Wilhelm K, Tami AE, Alini M (2010). Endothelial progenitor cells and mesenchymal stem cells seeded onto beta-TCP granules enhance early vascularization and bone healing in a critical-sized bone defect in rats. Tissue Eng Part A.

[14] Lockhart RA, Aronowitz JA, Dos-Anjos Vilaboa S (2017). Use of freshly isolated human adipose stromal cells for clinical applications. Aesthet Surg J.

[15] Bourin P, Bunnell BA, Casteilla L, Dominici M, Katz AJ, March KL (2013). Stromal cells from the adipose tissue-derived stromal vascular fraction and culture expanded adipose tissue-derived stromal/stem cells: a joint statement of the International Federation for Adipose Therapeutics and Science (IFATS) and the International Society for Cellular Therapy (ISCT). Cytotherapy.

[16] Maijub JG, Boyd NL, Dale JR, Hoying JB, Morris ME, Williams SK (2015). Concentration-dependent vascularization of adipose stromal vascular fraction cells. Cell Transplant.

[17] Zimmerlin L, Donnenberg VS, Pfeifer ME, Meyer EM, Péault B, Rubin JP (2010). Stromal vascular progenitors in adult human adipose tissue. Cytometry A.

[18] Laschke MW, Menger MD (2015). Adipose tissue-derived microvascular fragments: natural vascularization units for regenerative medicine. Trends Biotechnol.

[19] Weisberg SP, McCann D, Desai M, Rosenbaum M, Leibel RL, Ferrante AW (2003). Obesity is associated with macrophage accumulation in adipose tissue. J Clin Invest.

[20] Parra P, Serra F, Palou A (2010). Moderate doses of conjugated linoleic acid isomers mix contribute to lowering body fat content maintaining insulin sensitivity and a noninflammatory pattern in adipose tissue in mice. J Nutr Biochem.

[21] Frueh FS, Später T, Scheuer C, Menger MD, Laschke MW. Isolation of murine adipose tissue-derived microvascular fragments as vascularization units for tissue engineering. J Vis Exp. 2017;(122). 10.3791/55721 PMC5565147 28518106

[22] Stern R, McPherson M, Longaker MT (1990). Histologic study of artificial skin used in the treatment of full-thickness thermal injury. J Burn Care Rehabil.

[23] Böttcher-Haberzeth S, Biedermann T, Schiestl C, Hartmann-Fritsch F, Schneider J, Reichmann E (2012). Matriderm® 1 mm versus Integra® single layer 1.3 mm for one-step closure of full thickness skin defects: a comparative experimental study in rats. Pediatr Surg Int.

[24] Sorg H, Krueger C, Vollmar B (2007). Intravital insights in skin wound healing using the mouse dorsal skin fold chamber. J Anat.

[25] Sorg H, Krueger C, Schulz T, Menger MD, Schmitz F, Vollmar B (2009). Effects of erythropoietin in skin wound healing are dose related. FASEB J.

[26] Später T, Körbel C, Frueh FS, Nickels RM, Menger MD, Laschke MW (2017). Seeding density is a crucial determinant for the in vivo vascularisation capacity of adipose tissue-derived microvascular fragments. Eur Cell Mater.

[27] Später T, Frueh FS, Karschnia P, Menger MD, Laschke MW (2018). Enoxaparin does not affect network formation of adipose tissue-derived microvascular fragments. Wound Repair Regen.

[28] Mizuno H, Tobita M, Uysal AC (2012). Concise review: adipose-derived stem cells as a novel tool for future regenerative medicine. Stem Cells.

[29] Aronowitz JA, Ellenhorn JD (2013). Adipose stromal vascular fraction isolation: a head-to-head comparison of four commercial cell separation systems. Plast Reconstr Surg.

[30] Doi K, Tanaka S, Iida H, Eto H, Kato H, Aoi N (2013). Stromal vascular fraction isolated from lipo-aspirates using an automated processing system: bench and bed analysis. J Tissue Eng Regen Med.

[31] Granel B, Daumas A, Jouve E, Harlé JR, Nguyen PS, Chabannon C (2015). Safety, tolerability and potential efficacy of injection of autologous adipose-derived stromal vascular fraction in the fingers of patients with systemic sclerosis: an open-label phase I trial. Ann Rheum Dis.

[32] Guillaume-Jugnot P, Daumas A, Magalon J, Jouve E, Nguyen PS, Truillet R (2016). Autologous adipose-derived stromal vascular fraction in patients with systemic sclerosis: 12-month follow-up. Rheumatology (Oxford).

[33] Pilia M, McDaniel JS, Guda T, Chen XK, Rhoads RP, Allen RE (2014). Transplantation and perfusion of microvascular fragments in a rodent model of volumetric muscle loss injury. Eur Cell Mater..

[34] Laschke MW, Karschnia P, Scheuer C, Heß A, Metzger W, Menger MD (2018). Effects of cryopreservation on adipose tissue-derived microvascular fragments. J Tissue Eng Regen Med.

[35] Frueh FS, Menger MD, Lindenblatt N, Giovanoli P, Laschke MW (2017). Current and emerging vascularization strategies in skin tissue engineering. Crit Rev Biotechnol.

[36] Prins HJ, Schulten EA, Ten Bruggenkate CM, Klein-Nulend J, Helder MN (2016). Bone regeneration using the freshly isolated autologous stromal vascular fraction of adipose tissue in combination with calcium phosphate ceramics. Stem Cells Transl Med.

[37] Laschke MW, Menger MD (2017). Life is 3D: boosting spheroid function for tissue engineering. Trends Biotechnol.

[38] Laschke MW, Schank TE, Scheuer C, Kleer S, Schuler S, Metzger W (2013). Three-dimensional spheroids of adipose-derived mesenchymal stem cells are potent initiators of blood vessel formation in porous polyurethane scaffolds. Acta Biomater.

[39] van Dijk A, Naaijkens BA, Jurgens WJ, Nalliah K, Sairras S, van der Pijl RJ (2011). Reduction of infarct size by intravenous injection of uncultured adipose derived stromal cells in a rat model is dependent on the time point of application. Stem Cell Res.

[40] You D, Jang MJ, Kim BH, Song G, Lee C, Suh N (2015). Comparative study of autologous stromal vascular fraction and adipose-derived stem cells for erectile function recovery in a rat model of cavernous nerve injury. Stem Cells Transl Med.

[41] Frueh FS, Später T, Lindenblatt N, Calcagni M, Giovanoli P, Scheuer C (2017). Adipose tissue-derived microvascular fragments improve vascularization, Lymphangiogenesis, and integration of dermal skin substitutes. J Invest Dermatol.

[42] Später T, Frueh FS, Metzger W, Menger MD, Laschke MW (2018). In vivo biocompatibility, vascularization, and incorporation of Integra® dermal regenerative template and flowable wound matrix. J Biomed Mater Res B Appl Biomater.

[43] Koh YJ, Koh BI, Kim H, Joo HJ, Jin HK, Jeon J (2011). Stromal vascular fraction from adipose tissue forms profound vascular network through the dynamic reassembly of blood endothelial cells. Arterioscler Thromb Vasc Biol.

[44] Utzinger U, Baggett B, Weiss JA, Hoying JB, Edgar LT (2015). Large-scale time series microscopy of neovessel growth during angiogenesis. Angiogenesis.

[45] Ehrmantraut S, Laschke MW, Merkel D, Scheuer C, Willnecker V, Meyer-Lindenberg A (2010). Perioperative steroid administration inhibits angiogenic host tissue response to porous polyethylene (Medpor) implants. Eur Cell Mater.

[46] Hussain T, Schneider M, Summer B, Strieth S (2016). Pre-operative in vitro fibroblast coating of porous polyethylene compound grafts - cell survival in vivo and effects on biocompatibility. Biomed Mater Eng.

[47] Laschke MW, Menger MD (2016). The dorsal skinfold chamber: a versatile tool for preclinical research in tissue engineering and regenerative medicine. Eur Cell Mater..

[48] De Vriese AS, Verbeuren TJ, Vallez MO, Lameire NH, De Buyzere M, Vanhoutte PM (2000). Off-line analysis of red blood cell velocity in renal arterioles. J Vasc Res.

