# Supplementary figures and images for: Prevascularization of collagen-glycosaminoglycan scaffolds: stromal vascular fraction versus adipose tissue-derived microvascular fragments
Source: J Biol Eng. 2018 Nov 13;12:24. doi: 10.1186/s13036-018-0118-3 (PMC6234670; doi:10.1186/s13036-018-0118-3)

## Slide 1
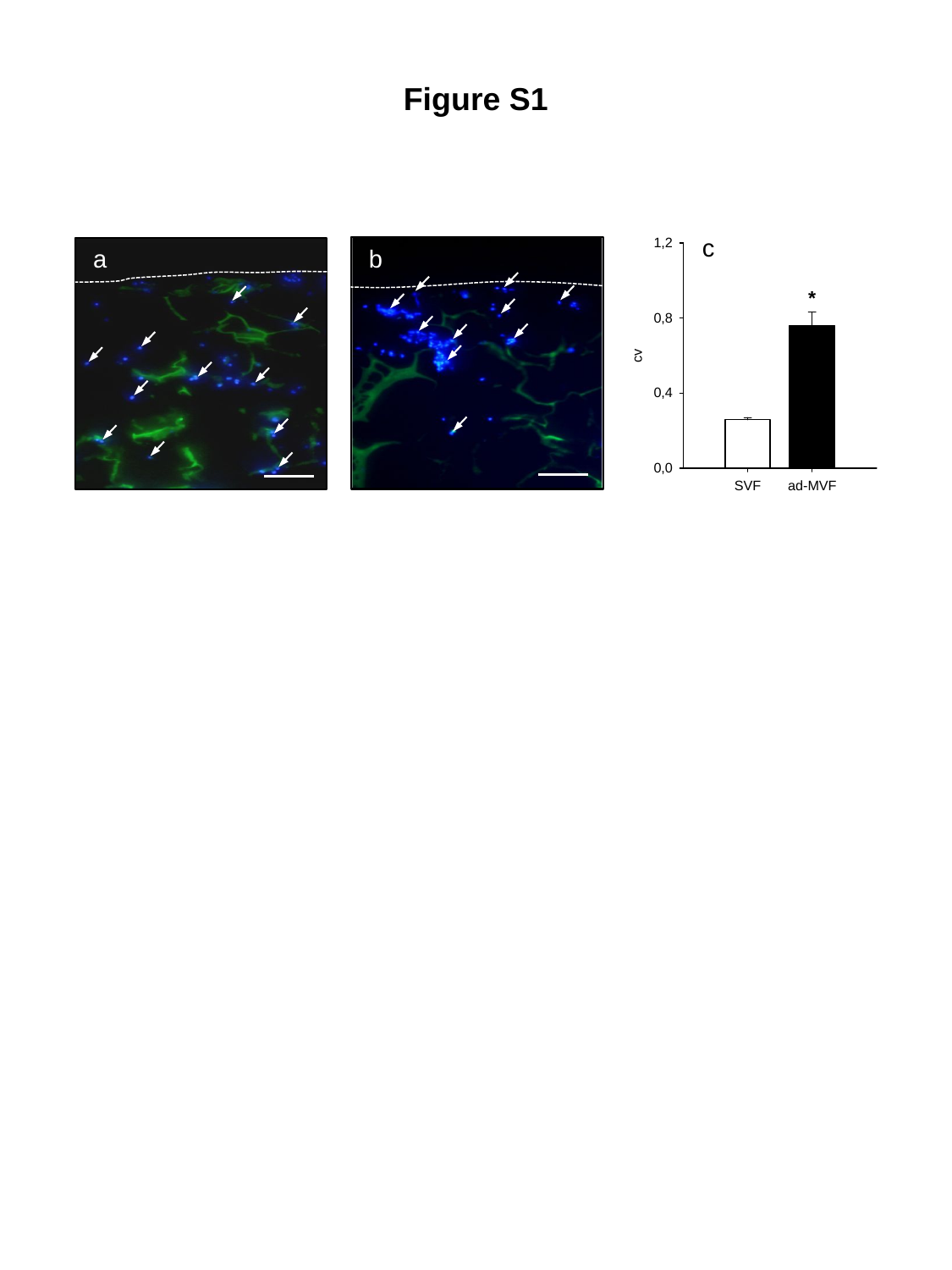

Figure S1
c
a
b

Supplement: Supplementary file 1 — Figure S1. Cell distribution within SVF- and ad-MVF-seeded scaffolds. a, b Detection of cell nuclei (arrows) within a SVF- (a) and an ad-MVF-seeded (b) Integra® scaffold (dotted lines = implant border; green signals = autofluorescence of the biomaterial). Scale bars: 150 μm. c Cv of SVF- (white bar, n = 4) and ad-MVF-seeded (black bar, n = 4) Integra® scaffolds directly after the seeding procedure, as assessed by histology. Means ± SEM. *p < 0.05 vs. SVF-seeded Integra® scaffolds. (PPTX 849 kb) [file 13036_2018_118_MOESM1_ESM.pptx]

## Slide 1
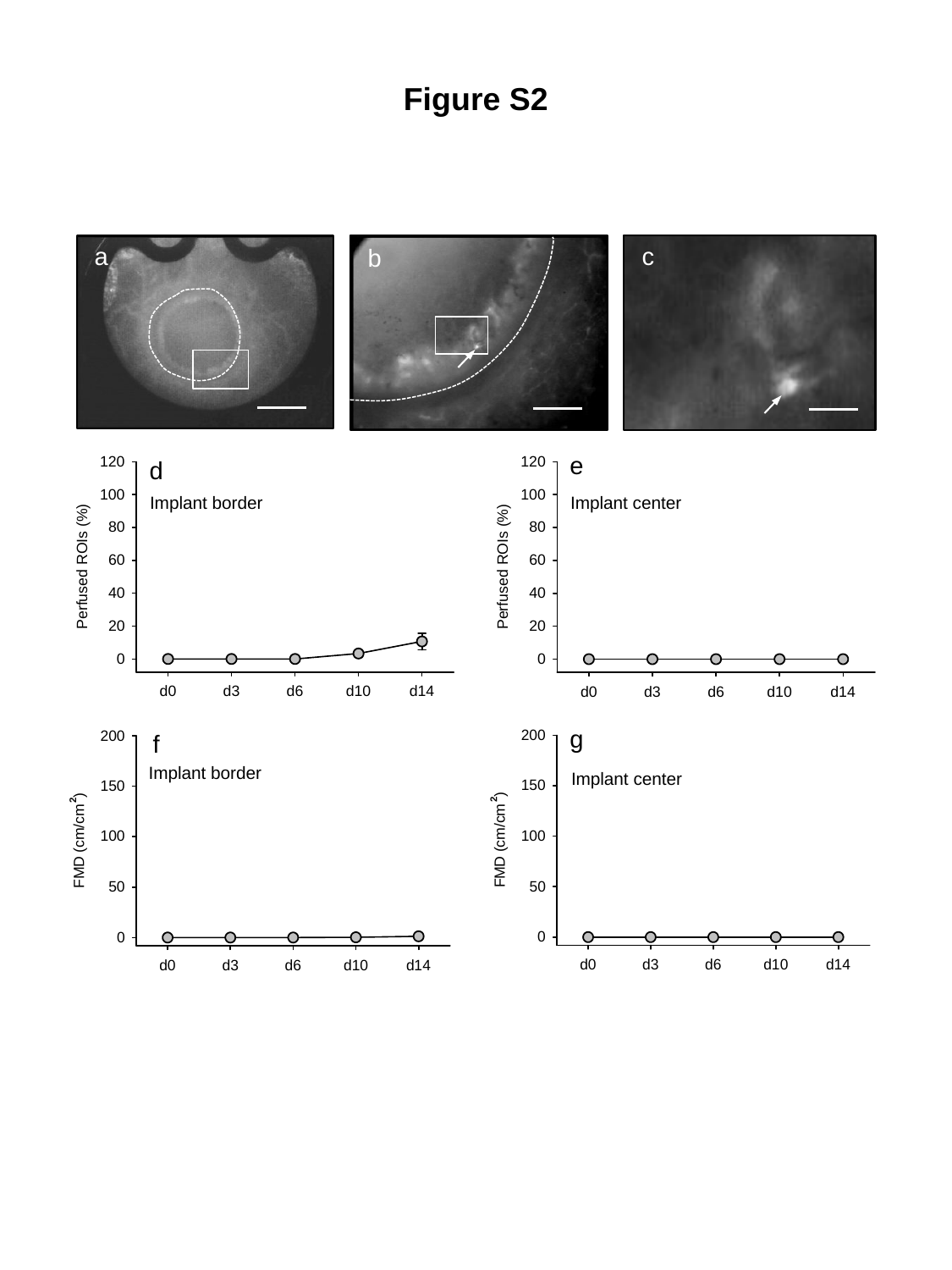

Figure S2
c
a
b
e
d
Implant border
Implant center
g
f
Implant border
Implant center

Supplement: Supplementary file 2 — Figure S2. Intravital fluorescence microscopy of implanted non-seeded scaffolds. a-c Intravital fluorescence microscopy (blue light epi-illumination with contrast enhancement by 5% FITC-labeled dextran) of a non-seeded Integra® scaffold on day 14 after implantation into a full-thickness skin defect within the dorsal skinfold chamber of a C57BL/6 recipient mouse (dotted lines = implant borders; arrows = perfused blood vessels; b, c = higher magnifications of inserts in a and b). Scale bars: a = 2.4 mm; b = 500 μm; c = 125 μm. d-g Perfused ROIs (d, e) and FMD (f, g) in the border (d, f) and center zones (e, g) of non-seeded Integra® scaffolds (grey circles, n = 4) on day 0, 3, 6, 10 and 14 after implantation, as assessed by intravital fluorescence microscopy. Means ± SEM. (PPTX 651 kb) [file 13036_2018_118_MOESM2_ESM.pptx]

## Slide 1
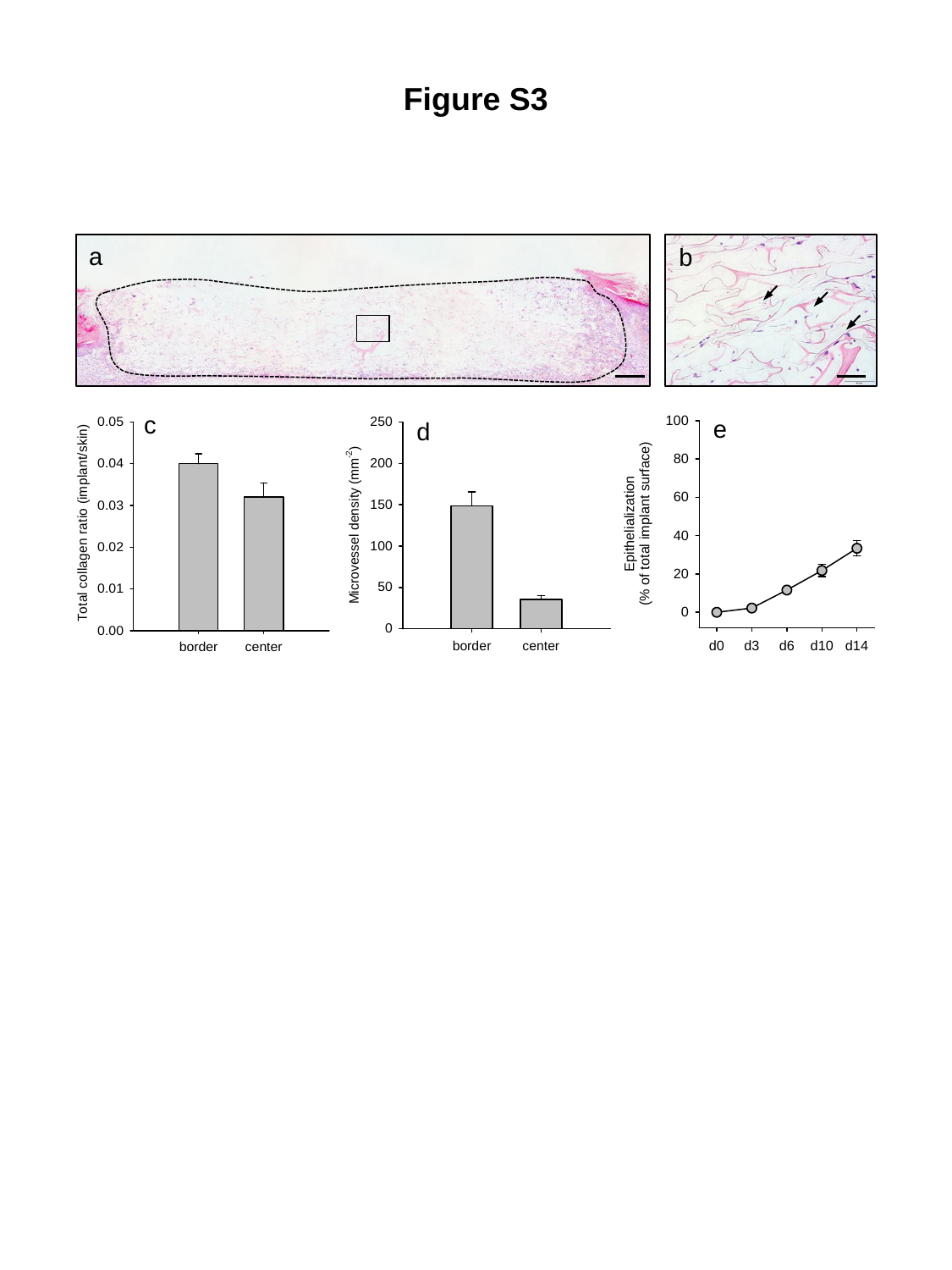

Figure S3
a
b
c
e
d

Supplement: Supplementary file 3 — Figure S3. Incorporation, vascularization and epithelialization of implanted non-seeded scaffolds. a, b HE-stained section of a non-seeded Integra® scaffold on day 14 after implantation into a full-thickness skin defect within the dorsal skinfold chamber of a C57BL/6 recipient mouse (broken line = implant; closed frame = center zone of the implant; b = higher magnification of closed frame in a; arrows = nuclei of individual cells). Scale bars: a = 260 μm; b = 40 μm. c Total collagen ratio in the border and center zones of non-seeded Integra® scaffolds (grey bars, n = 4) on day 14 after implantation, as assessed by histology. Means ± SEM. d Microvessel density in the border and center zones of non-seeded Integra® scaffolds (grey bars, n = 4) on day 14 after implantation, as assessed by immunohistochemistry. Means ± SEM. e Epithelialization of non-seeded Integra® scaffolds (grey circles, n = 4) on day 0, 3, 6, 10 and 14 after implantation, as assessed by trans-illumination microscopy. Means ± SEM. (PPTX 1734 kb) [file 13036_2018_118_MOESM3_ESM.pptx]
